# Comparison of the refractive prediction errors of artifcial intelligence formula with 3 conventional formulas and 11 combination methods in cataract surgery on eyes with short axial length

**DOI:** 10.5935/0004-2749.2023-0215

**Published:** 2024-09-16

**Authors:** Song-A Che, Mincheol Seong, Kookyoung Kim, Yong Woo Lee

**Affiliations:** 1 Kangwon National University Hospital, Kangwon National University School of Medicine, Chuncheon, Republic of Korea; 2 Guri Hanyang University Hospital, Hanyang University School of Medicine, Guri, Republic of Korea; 3 Konyang University Hospital, Konyang University School of Medicine, Daejeon, Republic of Korea

**Keywords:** Cataract, Lenses, intraocular, Axial length, eye, Refractive errors, Artificial intelligence

## Abstract

**Purpose:**

To compare the refractive prediction error of Hill-radial basis function 3.0 with those of 3 conventional formulas and 11 combination methods in eyes with short axial lengths.

**Methods:**

The refractive prediction error was calculated using 4 formulas (Hoffer Q, SRK-T, Haigis, and Hill-RBF) and 11 combination methods (average of two or more methods). The absolute error was determined, and the proportion of eyes within 0.25-diopter (D) increments of absolute error was analyzed. Furthermore, the intraclass correlation coefficients of each method were computed to evaluate the agreement between target refractive error and postoperative spherical equivalent.

**Results:**

This study included 87 eyes. Based on the refractive prediction error findings, Hoffer Q formula exhibited the highest myopic errors, followed by SRK-T, Hill-RBF, and Haigis. Among all the methods, the Haigis and Hill-RBF combination yielded a mean refractive prediction error closest to zero. The SRK-T and Hill-RBF combination showed the lowest mean absolute error, whereas the Hoffer Q, SRK-T, and Haigis combination had the lowest median absolute error. Hill-radial basis function exhibited the highest intraclass correlation coefficient, whereas SRK-T showed the lowest. Haigis and Hill-RBF, as well as the combination of both, demonstrated the lowest proportion of refractive surprises (absolute error >1.00 D). Among the individual formulas, Hill-RBF had the highest success rate (absolute error ≤0.50 D). Moreover, among all the methods, the SRK-T and Hill-RBF combination exhibited the highest success rate.

**Conclusions:**

Hill-radial basis function showed accuracy comparable to or surpassing that of conventional formulas in eyes with short axial lengths. The use and integration of various formulas in cataract surgery for eyes with short axial lengths may help reduce the incidence of refractive surprises.

## INTRODUCTION

Cataract surgery has evolved into a sophisticated refractive surgery, transcending its traditional role of treating vision loss from lens opacity. Despite extensive efforts to anticipate refractive errors in cataract surgery, eyes with an axial length (AL) beyond the normal range frequently encounter unexpected refractive errors postoperatively. Notably, the risk of such errors is increased in eyes with a shorter-than-normal AL^(1^,^2)^. Thus, accurate prediction of postoperative refractive error in eyes with a short AL remains a significant challenge for ophthalmologists performing cataract surgery.

Although many new-generation formulas have been developed, numerous primary clinics continue to use the third-generation SRK-T formula in the IOL master 500 (Carl Zeiss Meditec, Germany) owing to its cost and time efficiencies. This formula employs two key parameters, namely, corneal curvature and AL^(3)^. In 2008, Gavin et al. suggested that the Hoffer Q formula is more accurate than SRK-T for eyes with a short AL^(4)^. However, recent studies have reported that there is no significant difference in accuracy between Hoffer Q and SRK-T^(5^,^6^,^7)^. The Haigis formula^(8)^, which includes anterior chamber depth (ACD) as an additional variable, is also widely used in the IOL Master 500.

Recently, many advanced formulas, such as Barrett Universal II, Kane, and Olsen, have been developed and utilized. In addition, the Hill-RBF formula uses artificial intelligence for pattern recognition to determine lens power. It was initially developed using data from 3,445 eyes; in 2018, version 2.0 was released, with data from 12,419 eyes added^(9^,^10^,^11)^. In version 3.0, the size of the database was further expanded.

The aforementioned formulas can be used to determine intraocular lens (IOL) power if biometry is outside of the normal range to reduce the occurrence of unexpected large refractive error.

This study aimed to compare and analyze the accuracy of refractive error predictions made by the SRK-T, Hoffer Q, Haigis, and Hill-RBF 3.0 formulas in cataract surgery on eyes with short AL as well as investigate the outcomes of combining two or more of these formulas in such cases.

## METHODS

The study adhered to the principles of the Declaration of Helsinki and was approved by the Ethics Review Committee of Kim’s Eye Hospital. The study included patients who underwent simple cataract surgery and posterior chamber IOL implantation with the use of Tecnis ZCB00 monofocal IOL (AMO, USA) in eyes with an AL ≤22.00 mm in the IOLMaster 500 (version 7.5, Carl Zeiss Meditec, Germany) in Kim’s Eye Hospital and Guri Hanyang University Hospital from August 2016 to December 2018. All surgeries were performed by two cataract specialists (MCS, YWL). The patients underwent a comprehensive ophthalmologic examination, including pre- and postoperative visual acuity tests, noncontact tonometry, and mydriatic fundus examination. One month postoperatively, best-corrected visual acuity and manifest refraction tests were conducted.

Eyes with ophthalmologic diseases that could affect AL measurement, such as corneal disease, glaucoma, and retinal disease, were excluded. Cases with complications that could potentially affect refractive error measurements, such as zonulysis and posterior capsular rupture during surgery, were also excluded. Furthermore, patients with corrected visual acuity <0.8 on the Snellen chart 1 month postoperatively were excluded. To minimize bias, only the right eye of each patient was included^(12)^.

The target refractive error (TRE) calculated using the Hoffer Q, SRK-T, and Haigis formulas integrated into the IOLMaster 500, along with the Hill-RBF formula available on the website (http://rbfcalculator.com/), was compared with the postoperative spherical equivalent (PSE) 1 month postoperatively. The A-constants utilized in these formulas were standardized based on the recommendations on the User Group for Laser Interference Biometry (ULIB) website (http://ocusoft.de/ulib/), optimized for compatibility with the IOLMaster 500.

The TRE of the four individual formulas and 11 combination methods were calculated. For the combination methods, an average was derived from two or more individual formulas. The intraclass correlation coefficient (ICC) was used to evaluate the agreement with the PSE. The refractive prediction error (RPE) was defined as the difference between the TRE of each method and the PSE, with the absolute error (AE) calculated from the absolute value of the RPE. Then, the mean AE (MAE) and median AE (MedAE) were determined. Multiple regression analysis was employed to identify the factors affecting the RPE. Furthermore, the AE was divided by 0.25 diopter (D), and the proportion of the eyes was calculated. “Success” was defined as AE ≤0.50 D and “surprise” as AE >1.00 D. In addition, Barrett Universal II and Kane were compared with Hill-RBF. Statistical analyses were conducted using the SPSS software (version 26.0, IBM Corp., NY, USA).

## RESULTS

Among the 278 eyes with an AL ≤22 mm, 87 from 87 patients who met the inclusion criteria were included in the study. The mean age of the patients was 65.57 ± 9.47 years, and 11 and 76 of them were men and women, respectively. Table 1 summarizes the corrected visual acuity, refractive error, diopters of IOL used, and ocular measurements pre- and postoperatively.

**Table 1:** Patient demographics and ocular biometry (n=87)

Characteristics	Mean ± standard deviation	Range
Age (years)	65.57 ± 9.47	19.00-82.00
Sex (n, %)		
Male	11 (12.6)	
Female	76 (87.4)	
Hypertension (n, %)	43 (49.4)	
Diabetes mellitus (n, %)	21 (24.1)	
IOL power (D)	26.68 ± 1.51	23.00-32.00
Preop IOP (mmHg)	14.91 ± 2.84	9.00-22.00
Preop CDVA (LogMAR)	0.32 ± 0.28	0-1.00
Preop sphere (D)	1.78 ± 2.40	-3.50-7.50
Preop cylinder (D)	-0.82 ± 0.69	-2.75-0
Preop SE (D)	1.36 ± 2.34	-4.25-6.75
Postop IOP (mmHg)	12.95 ± 2.71	8.00-19.00
Postop CDVA (LogMAR)	0.04 ± 0.04	0-0.10
Postop sphere (D)	0.07 ± 0.58	-1.50-1.50
Postop cylinder (D)	-0.88 ± 0.66	-3.00-0
Postop SE (D)	-0.37 ± 0.48	-1.75-0.88
Axial length (mm)	21.40 ± 0.40	20.31-22.00
SNR	159.51 ± 107.27	1.00-459.50
Flat K (D)	45.33 ± 1.29	42.13-48.42
Steep K (D)	46.20 ± 1.30	43.49-49.49
Mean K (D)	45.77 ± 1.27	43.03-48.96
ACD (mm)	2.63 ± 0.33	2.06-3.51

IOL= intraocular lens; D= diopters; IOP= intraocular pressure; CDVA= corrected distance visual acuity; LogMAR= logarithm of the minimum angle of resolution; SE= spherical equivalent; SNR= signalnoise ratio; K= keratometry; ACD, anterior chamber depth.

The mean RPE showed the most myopic error in Hoffer Q (average: -0.330 ± 0.469 D, range: -1.525–0.775 D), followed by SRK-T (average: -0.088 ± 0.505 D, range: -1.220–1.535 D), Hill-RBF (average: -0.075 ± 0.450 D, range: -1.395–0.865 D), and Haigis (average: 0.043 ± 0.508 D, range: -1.485–1.000 D). Among all the methods, the Haigis and Hill-RBF combination yielded an RPE closest to zero (average: -0.016 ± 0.472, range: -1.440–0.885 D) (Table 2).

**Table 2:** Values of the refractive prediction error (RPE), mean absolute error (MAE), median absolute error (MedME), and intraclass correlation coefficient (ICC) of the 4 formulas (SRK-T, Hoffer Q, Haigis, and Hill-RBF) and 11 combination methods in eyes with short axial lengths (n=87) (the average value of the methods connected by the + sign was used)

Formula	RPE (diopters) (mean ± SD, range)	MAE (diopters) (mean ± SD, range)	MedME	ICC
Hoffer Q	-0.330 ± 0.469 (-1.525-0.775)	0.442 ± 0.364 (0.000-1.525)	0.350	0.363
SRK-T	-0.088 ± 0.505 (-1.220-1.535)	0.398 ± 0.320 (0.010-1.535)	0.310	0.220
Haigis	0.043 ± 0.508 (-1.485-1.000)	0.399 ± 0.315 (0.000-1.485)	0.345	0.463
Hill-RBF	-0.075 ± 0.450 (-1.395-0.865)	0.353 ± 0.287 (0.000-1.395)	0.305	0.532
Hoffer Q + SRK-T	-0.209 ± 0.474 (-1.285-1.155)	0.387 ± 0.343 (0.005-1.285)	0.340	0.305
Hoffer Q + Haigis	-0.143 ± 0.472 (-1.505-0.845)	0.373 ± 0.320 (0.005-1.505)	0.285	0.439
Hoffer Q + Hill-RBF	-0.202 ± 0.448 (-1.460-0.820)	0.371 ± 0.321 (0.000-1.460)	0.265	0.470
SRK-T + Haigis	-0.022 ± 0.469 (-1.195-1.200)	0.368 ± 0.290 (0.005-1.200)	0.290	0.401
SRK-T + Hill-RBF	-0.081 ± 0.454 (-1.145-1.200)	0.351 ± 0.297 (0.005-1.200)	0.275	0.423
Haigis + Hill-RBF	-0.016 ± 0.472 (-1.440-0.885)	0.369 ± 0.292 (0.010-1.440)	0.350	0.504
Hoffer Q + SRK-T + Haigis	-0.125 ± 0.464 (-1.268-1.058)	0.360 ± 0.316 (0.000-1.268)	0.257	0.394
Hoffer Q + Haigis + Hill-RBF	-0.121 ± 0.460 (-1.468-0.835)	0.360 ± 0.309 (0.002-1.468)	0.263	0.477
Hoffer Q + SRK-T + Hill-RBF	-0.164 ± 0.453 (-1.238-1.058)	0.358 ± 0.322 (0.005-1.238)	0.283	0.410
SRK-T + Haigis + Hill-RBF	-0.040 ± 0.457 (-1.237–-1.088)	0.354 ± 0.289 (0.007-1.237)	0.303	0.456
Hoffer Q + SRK-T + Haigis + Hill-RBF	-0.112 ± 0.456 (-1.300-1.010)	0.354 ± 0.306 (0.005-1.300)	0.268	0.440

The SRK-T and Hill-RBF combination (average: 0.351 ± 0.297 D, range: 0.005–1.200 D) yielded the lowest

MAE among all the methods. Among the individual formulas, Hill-RBF had the lowest MAE (average: 0.353 ± 0.287 D, range: 0.000–1.395 D). The MAEs were 0.442 ± 0.364 D for Hoffer Q, 0.398 ± 0.320 D for SRK-T, and 0.399 ± 0.315 D for Haigis. The combination methods typically yielded lower MAEs than the individual formulas, except for Hill-RBF. All the combination methods yielded an MAE of <0.39 D. The lowest MedAE was observed in the Hoffer Q, SRK-T, and Haigis combination. Among the individual formulas, Hill-RBF had the lowest MedAE (Table 2).

The Hill-RBF formula exhibited the highest agreement between TRE and PSE (ICC=0.532). Among the 11 combination methods, the Haigis and Hill-RBF combination had the highest ICC, which was 0.504 (Table 2).

**Figure 1:**
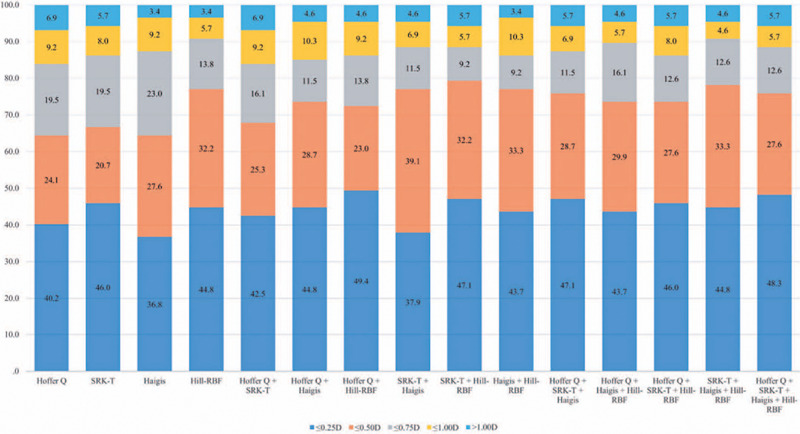
Percentages of eyes within the specified diopter ranges of absolute errors across varioudsformulas (SRK-T, Hoffer Q, Haigis, and Hill-RF,) and combination methos) in eyes with short axial lengths n=87).

The AE was segmented into 0.25-D increments to calculate the ratio for each formula. The proportions of eyes with AE ≤0.50 D were 64.4% for Hoffer Q, 66.7% for SRK-T, 64.4% for Haigis, and 77.0% for Hill-RBF. The SRK-T and Hill-RBF combination had the highest success rate (79.3%) (Figure 1).

Among the individual formulas, Haigis and Hill-RBF had the lowest refractive surprise rate (both 3.4%). The Haigis and Hill-RBF combination also showed the lowest refractive surprise rate (3.4%). Contrarily, SRK-T and Hoffer Q had refractive surprise rates of 5.7% and 6.9%, respectively (Figure 1).

Multiple regression analysis conducted to assess the factors affecting the RPE revealed that AL had a significant effect on the RPE in Hoffer Q (RPE_HofferQ_ = 6.165–0.304 * AL, p=0.016, *R*^2^=0.067) and the IOL power had a significant effect on the RPE in SRK-T (RPE_SRK-T_ = -3.487 + 0.127 * IOL power, p<0.001, *R*^2^=0.145). In addition, steep keratometry (K) and ACD exerted significant effects on the RPE in Haigis (RPE_Haigis_ = -7.521 + 0.181 * steep K – 0.304 * ACD, p<0.001, *R*^2^=0.235), and the average K significantly impacted the RPE in Hill-RBF (RPE_Hill-RBF_ = -4.497 + 0.097 * average K, p=0.011, *R*^2^=0.074) (Table 3).

**Table 3:** Factors influencing the predicted refractive errors of four individual formulas (Hoffer Q, SRK-T, Haigis, and Hill-RBF) in eyes with short axial lengths

	Factors	Regression formulas
Hoffer Q	Axial length (AL)	6.165 - 0.304 × AL (*P*=0.016, *R*^2^=0.067)
SRK-T	Intraocular lens (IOL) power	-3.487 + 0.127 × IOL power (*P*<0.001, *R*^2^=0.145)
Haigis	Steep keratometry (steep K) Anterior chamber depth (ACD)	-7.521 + 0.181 × steep K - 0.304 × ACD (*P*<0.001, *R*^2^=0.235)
Hill-RBF	Mean keratometry (mean K)	-4.497 + 0.097 × mean K (*P*=0.011, *R*^2^=0.074)

In the comparison of recent generation formulas, the RPE exhibited a myopic trend in the following order: Kane, Barrett Universal II, and Hill-RBF. Barrett Universal II showed the lowest median absolute error (MedME) (0.290 D). However, after adjusting the mean to zero, Hill-RBF showed the lowest MedME value (0.295 D) (Table 4).

**Table 4:** Refractive prediction error (RPE), mean absolute error (MAE), median absolute error (MedME), and intraclass correlation coefficient (ICC) of new-generation formulas (Hill-RBF, Barrett Universal II, and Kane) in eyes with short axial lengths (n=87)

Formula	RPE (diopters) (mean ± SD, range)	MAE (diopters) (mean ± SD, range)	MedME	ICC	After adjusting the mean RPE to Zero	
					MAE (diopters) (mean ± SD, range)	MedME
Hill-RBF	-0.075 ± 0.450 (-1.395-0.865)	0.353 ± 0.287 (0.000-1.395)	0.305	0.532	0.362 ± 0.264 (0.025-1.320)	0.295
Barrett Universal II	-0.116 ± 0.456 (-1.495-0.875)	0.360 ± 0.300 (0.010-1.495)	0.280	0.524	0.364 ± 0.272 (0.006-1.379)	0.299
Kane	-0.206 ± 0.474 (-1.400-1.025)	0.392 ± 0.334 (0.000-1.400)	0.290	0.377	0.377 ± 0.284 (0.006-1.231)	0.316

## DISCUSSION

Although the prediction of the effective lens position has become increasingly precise, refractive errors after cataract surgery in eyes with a short AL remains unsatisfactory^(13^,^14)^. Although many new-generation formulas have been developed, numerous surgeons continue to use traditional formulas as they are readily available through the IOLMaster 500 (Carl Zeiss Meditec, Germany), a widely used biometric device.

Several previous studies have reported that Hoffer Q is more accurate for eyes with a short AL^(4^,^15^,^16)^. However, recent evidence indicates that there is no significant difference in surgical outcomes among the different formulas^(5^,^6^,^17)^.

The Hill-RBF formula uses artificial intelligence to predict the IOL power by analyzing the patterns within a vast dataset of thousands of cases rather than a theoretical formula. With the release of Hill-RBF 3.0 in 2020, the range of supported IOLs was expanded to include biconvex lenses with powers from +34.0 to +6.0 D and meniscus lenses with powers ranging from +5.0 to -5.0 D, significantly reducing the occurrence of “out-of-bound” errors.

In a previous study focusing on the performance of Hill-RBF in eyes with short ALs, Gokce et al. found an average predicted refractive error of +0.05 ± 0.47 D and an MAE of 0.36 ± 0.30 D when using personalized values of the A-constant^(17)^. There was no statistically significant difference from those of Hoffer Q and Haigis when the mean was adjusted to zero. They also reported that the proportion of RPE within ±0.5 D was slightly higher in Hill-RBF (70.9%) than in Haigis (68.6%) and Hoffer Q (64.0%), but the difference was not statistically significant. Notably, these findings may be related to the original version of Hill-RBF (version 1.0, released in April 2017), which was based on a database of approximately 3,212 cases.

In this study, only one IOL type of was included, and the IOL constant was chosen based on ULIB without personalization. The mean RPE (-0.075 ± 0.450 D), MAE (0.353 ± 0.287 D), and probability of obtaining RPE ≤0.5 D (77.0%) showed improved results.

In another study conducted by Roberts et al.^(10)^, which analyzed the performance of Hill-RBF, the MAE was 0.37 ± 0.33 D in eyes with an AL of <22 mm, which was lower than those in other formulas. Furthermore, the proportion of eyes with significant refractive surprises (>1.00 D) was only 4.8% (1 of 21 eyes), which was comparable to that observed with Barrett Universal II. In our study, Hill-RBF yielded the lowest MAE (0.353 ± 0.287 D), and only 3 out of 87 eyes (3.4%) had a refractive error >1 D.

Although the new-generation formulas may not significantly improve the mean RPE, they potentially offer a notable advantage in reducing the probability of unexpected severe outcomes. Furthermore, because previous studies have used the 2017 version of Hill-RBF, some of the improved results may be explained by Hill-RBF 3.0, which was released in 2020.

Most previous studies compared the errors through mean analysis to find statistical significance. However, it may be difficult to obtain significant differences when analyzing extremely small values (<0.25 D). In this study, ICC was used to compare the agreement of two values. In general, ICC is used to analyze repeatability and reliability^(18^,^19)^. ICC has been suggested to be more appropriate in analyzing the agreement by obtaining the same result in the same eye using different formulas. In our findings, the ICC was 0.532 in the Hill-RBF formula, which was much higher than those in other formulas, and the Haigis and Hill-RBF combination had the second-highest ICC (0.504). These results indicate that the agreement between the TRE and postoperative refractive error was most pronounced in Hill-RBF. The mean RPE and MAE of the combination methods were also compared. Several surgeons compare two or more formulas to decide the diopters of IOL before performing surgery. However, there is no study comparinging the results of the combination methods.

In this study, the Haigis and Hill-RBF combination yielded an RPE closest to zero among all the methods. The MAE was found to be the lowest in the SRK-T and Hill-RBF combination method, and the combinations of two or more formulas tended to have lower MAE. The highest success rate (AE ≤0.5 D) was observed in the SRK-T and Hill-RBF combination (79.3%); furthermore, all the combination methods displayed a success rate >70%, except for the Hoffer Q and SRK-T combination (Figure 1). These findings suggest that the combinations of two or more formulas further improve surgical outcomes in eyes with a short AL, where the differences in refractive errors can be significant.

In addition, our study findings indicated a higher proportion of women among the subjects with short ALs. This observation is consistent with that of a previous study showing that the ALs were shorter in women^(20)^. Although further investigation is warranted, this sex difference in ALs suggests that sex plays a role in IOL calculations.

The limitations of this study were that it was difficult to find patients undergoing cataract surgery with eyes having short ALs without any other complications, particularly glaucoma. Therefore, two surgeons conducted the surgeries. Furthermore, although recent studies have used advanced devices that can measure more diverse elements, such as IOLMaster 700 (Carl Zeiss Meditec, Jena, Germany) and Lenstar (Haag-Streit AG, Koeniz, Switzerland), our study relied on the IOLMaster 500, which has limitations in analyzing various factors and formulas. Larger-scale studies incorporating a more diverse range of factors and a greater number of cases are warranted to improve the robustness and applicability of these findings.

This study evaluated the refractive outcomes in eyes with short ALs using a combination of existing formulas and the Hill-RBF formula. As expected, Hill-RBF 3.0 exhibited higher accuracy in eyes with short AL than the traditional formula. Furthermore, it was comparable with any of the latest-generation formulas in terms of stability. Our analysis revealed that combining both the Hill-RBF and SRK-T formulas could lead to improved refractive outcomes. These results may help obtain more accurate prediction results in cataract surgery as a refractive procedure that currently requires greater sophistication.
